# Comparative analysis of metabolite signatures and hypoglycemic effects in Toona sinensis leaves processed by two distinct methods

**DOI:** 10.1016/j.fochx.2025.102821

**Published:** 2025-07-20

**Authors:** Guohuo Wu, Shuolei Xu, Zhaoyun Chen, Li Wu, Wenli Fan, Xiaoyun Wu, Mengyu Li, Changqing Qu, Yuntao Ji

**Affiliations:** a Engineering Technology Research Center of Anti-Aging Chinese Herbal Medicine of Anhui Province, School of Biology and Food Engineering, Fuyang Normal University, Fuyang, China; b The Second People's Hospital of Fuyang City, Fuyang, China

**Keywords:** Toona sinensis, Processing techniques, Metabolomics, Characteristic compounds, Hypoglycemic effect

## Abstract

The tender buds of “Heiyouchun” (*Toona sinensis*), a traditional Chinese specialty variety, are highly favored as a unique woody vegetable. However, the mature leaves exhibit distinct bitterness and lack aromatic compounds, resulting in low resource utilization. This study innovatively developed toon leaf tea (TLT) using black tea processing techniques. Through integrated metabolomic analysis and health benefit evaluation, we systematically compared the differences in chemical composition and hypoglycemic effect between TLT and traditionally dried mature toon leaves (TOL). Metabolomic analysis revealed that the two processing methods induced significant changes in 41 metabolites. Compared with TOL, TLT demonstrated significant enrichment in functional components including flavonoids (e.g., quercetin derivatives), limonoids, and amino acids, along with a marked reduction in phenolic acids and organic acids. The synergistic effects of these components effectively improved product flavor and enhanced bioactivity. In vitro and in vivo experiments confirmed that the α – glucosidase/α – amylase inhibitory activity of TLT and the hypoglycemic effect on diabetes model mice are significantly better than that of TOL. This study not only elucidates the transformation patterns of chemical constituents in mature toon leaves under different processing methods but also provides a scientific basis for the resource utilization of mature toon leaves.

## Highlights

• A total of 41 differential metabolites were identified between toon leaf (TOL) and toon leaf tea (TLT).
• TLT vs. TOL showed enriched flavonoids, limonoids, and amino acids, with reduced phenolic/organic acids.
• Black tea processing boosts hypoglycemic efficacy in toon leaves compared to direct drying.

## 1 Introduction

Chinese toona (*Toona sinensis* (A. Juss) Roem) is a valuable multifunctional tree species with a long cultivation history, wide geographical distribution, and remarkable adaptability (Zhao et al., 2024). As a species integrating food, medicinal, and material applications, it demonstrates significant potential for environmental beautification and resource utilization. The buds and young leaves of toon are particularly noteworthy as nutrient-dense vegetables, containing essential amino acids, vitamins, and polyunsaturated fatty acids (Jiang et al., 2019). Contemporary pharmacological studies have further revealed toon diverse bioactivities, including antidiabetic, antioxidant, anti-inflammatory, and hepatoprotective effects (Peng et al., 2019; Zhao et al., 2024).

The unique flavor profile and nutritional composition of toon's tender parts have driven their application in fermented sauces and functional food products (Jiang et al., 2019; Su et al., 2020). However, mature toon leaves, characterized by their pronounced bitterness and limited aromatic compounds, remain underutilized, leading to significant resource waste. In some regions of China, mature toon leaves are often dried directly and used for brewing tea, which is believed to possess auxiliary hypoglycemic effects (Wang et al., 2008). Numerous studies have substantiated the hypoglycemic activity of toon leaves (Hsieh et al., 2012; Yang et al., 2003). - Notably, Liu et al. (2015) demonstrated that toon leaf can ameliorate insulin resistance through the AMPK and PPARγ pathways. Nevertheless, the poor taste of directly dried toon leaves limits their popularity, severely restricting their resource utilization efficiency.

To address this limitation, the present study proposes an innovative approach by adapting black tea processing techniques to develop a novel toon leaf tea (TLT) product. Unlike green tea or oolong tea processing technologies that have stringent requirements for leaf tenderness and dark tea processing that has a long cycle, black tea processing technology is more suitable for processing mature toon leaves. Toon leaves are rich in secondary metabolites, and the application of black tea processing techniques may significantly alter their phytochemical composition and corresponding bioactivities. This observation is consistent with findings from the application of tea processing techniques to other plant materials (Wang et al., 2019). For instance, studies have indicated that processing mulberry leaves using black tea techniques can elevate their flavonoid content and enhance acetylcholinesterase inhibitory activity (Panyatip et al., 2022). Additionally, Chen et al. reported that processing coffee leaves using black tea processing methods can enhance the immunomodulatory activity of Raw 264.7 cells (Chen et al., 2018). The application of black tea processing methods to mature toon leaves not only enhances the product's flavor but also facilitates the biotransformation of bioactive compounds. Nonetheless, the specific alterations in the chemical composition and bioactivity of TLT, in comparison to traditionally directly dried mature toon leaves (TOL), remain to be fully elucidated and warrant further investigation.

This study focuses on *T. sinensis* var. ‘Heiyouchun’, a historically significant cultivar from Taihe County, China. Using untargeted metabolomics coupled with chemometric analysis, we systematically compared the chemical profiles between TLT and TOL, and evaluated the glycemic lowering effect by measuring α-amylase and α-glucosidase inhibitory activity in vitro, and glucose tolerance in high-fat diet/streptozotocin (HFD/STZ)-induced type 2 diabetes mellitus (T2DM) mice. The findings elucidate the metabolic foundation of TLT's functional attributes while advancing sustainable utilization strategies for mature toon leaves.

## 2 Material and methods

### 2.1 Chemicals

The chemical reference standards for rutin, quercetin, kaempferol, and gallic acid were sourced from Chem Faces (Wuhan, China). Analytical-grade solvents including acetonitrile, methanol, and formic acid, alongside glucose reagents, were obtained from Sigma-Aldrich (MO, USA). Enzymatic substrates *p*-Nitrophenyl-α-D-glucopyranoside (pNPG), α-glucosidase and α-amylase were provided by Yuanye Biotechnology (Shanghai, China). A commercial glycated serum protein (GSP) detection kit was acquired from Nanjing Jiancheng Bioengineering Institute (Nanjing, China). All additional chemical compounds employed in this study met analytical specifications or the highest available commercial purity standards.

### 2.2 Manufacturing of TLT and TOL

**Fig. 1:**
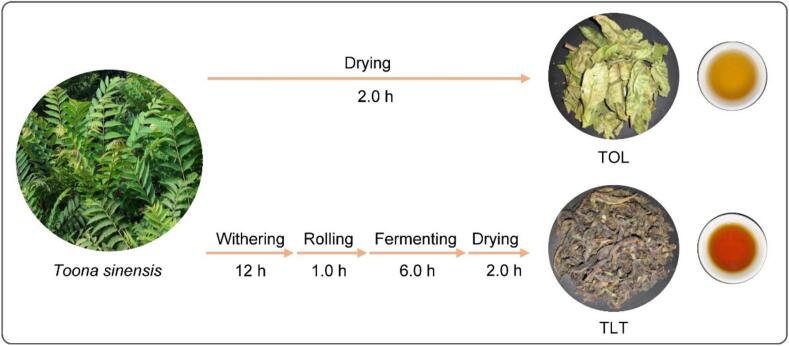
Two distinct processing procedures for *Toona Sinensis* leaves.

*T. sinensis* var. ‘Heiyouchun’ is cultivated at the Taihe County nursery in Anhui Province, China. Mature leaves should be systematically harvested from the seventh node of each branch in May, counting acropetally from the shoot apex. The experimental design included five biological replicates, with each replicate comprising mature leaves pooled from three independent *T. sinensis* trees. A total of 1 kg of leaves was collected from each tree, yielding 15 kg of raw material for the study. The harvested mature leaves are processed in two ways (Fig. 1): one portion is crafted using the black tea processing method to create toon leaf tea (TLT). Briefly, mature toon leaves undergo 12 h of indoor withering, followed by 1 h of rolling, 6 h of fermentation, drying at 110 °C for 2 h, and then cooling for 30 min to obtain TLT samples. The other portion of mature toon leaves is directly dried at 110 °C for 2 h, then cooled for 30 min to obtain toon leaves (TOL) samples. The resulting samples are frozen and stored at −20 °C, and uniformly ground using a mill for subsequent analysis.

### 2.3 Sample preparation for UHPLC-Orbitrap-MS/MS analysis

Sample extraction was performed following a previously established method (Li et al., 2024) using a 70 % methanol aqueous solution. Briefly, the five samples per group were extracted with 70 % aqueous methanol and sonicated for 30 min. Following that, 2 mL of the supernatant were collected, and the methanol was completely evaporated using nitrogen gas. The dried residue was then dissolved in 2 mL of pure water and loaded onto an activated Sep-Pak Vac C18 extraction column (Waters, Ireland). The column was rinsed with 1 mL of pure water, followed by a wash with 2 mL of pure methanol. After centrifugation at 12,000 r/min for 10 min, 400 μL of the supernatant were collected. This supernatant was dried completely using nitrogen gas, and the residue was dissolved in 400 μL of 70 % methanol. The solution was centrifuged again at 12,000 r/min for 10 min, and the resulting supernatant was harvested for further analysis. Quality control (QC) samples were prepared by mixing equal volumes (20 μL each) of each sample to monitor system stability during untargeted metabolomics analysis.

### 2.4 UHPLC-Orbitrap-MS/MS analysis

Chromatographic analysis was performed using an Ultimate HPLC system (Thermo Fisher Scientific, Waltham, MA, USA). Samples were separated on an ACQUITY HSS T3 column (100 mm × 2.1 mm, 1.8 μm; Waters, Milford, MA, USA). The mobile phase consisted of (A) 0.1 % formic acid in water and (B) 0.1 % formic acid in acetonitrile, with a flow rate of 0.30 mL/min under gradient elution conditions as follows: 0–5 min, 97 % A; 5–11 min, 92–70 % A; 11–20 min, 70–20 % A; 20–21 min, 20–5 % A; 21–27 min, 5 % A; 28–34 min, 97 % A.

Mass spectrometric detection was performed using a Q Exactive Focus Orbitrap MS system (Thermo Fisher Scientific, USA) equipped with a heated electrospray ionization (HESI) source. Mass spectra were acquired separately in positive and negative ionization modes with a mass range of 100–1000 *m*/*z*, using spray voltages of 3.5 kV and 3 kV, respectively. The capillary and source temperatures were maintained at 350 °C and 360 °C, respectively, with sheath gas (N2) and auxiliary gas (N2) pressures set at 50 psi and 10 psi, respectively. Sodium trifluoroacetate was used for accurate mass calibration. TIC overlay chromatograms of QC samples were shown in Fig. S1.

### 2.5 Data analysis for untargeted metabolomics

For mass spectra data analysis, raw UHPLC-Orbitrap-MS/MS data were processed via XCMS Online (http://xcmsonline.scripps.edu/) for peak deconvolution and alignment. The primary parameters for data processing were configured as follows: an ion intensity threshold of 100,000, an ion *m*/*z* tolerance of 20 ppm, a retention time range of 0–30 min, and an ion retention time tolerance of 0.5 min. All other parameters were set to their default values. For data normalization, the area method was employed to account for intensity variations. Following these preprocessing steps, the data matrix was constructed and exported to an Excel table, which included key variables such as retention time (RT), m/z value, and the normalized ion intensity. Principal Component Analysis (PCA) and Partial Least Squares-Discriminant Analysis (PLS-DA) were executed in SIMCA-P 13.0 (Umetrics, Sweden). Preprocessing for PCA and PLS-DA involved filtering metabolites with a relative standard deviation (RSD) > 30 % in QC samples to ensure data reliability. Data normalization using total sample area minimized systematic ion intensity variations and batch effects, enhancing multivariate analysis robustness. To ensure model validity and mitigate overfitting risks, permutation testing involving 200 iterative validations was implemented. Critical model parameters were derived from the PLS-DA framework, encompassing Variable Importance in Projection (VIP) scores, fold-change (FC = TLT/TOL) ratios, and statistical probability values (*p*-values).

Metabolite differentiation between TLT and TOL groups was established through multi-criteria screening: relative standard deviation (RSD) < 30 %, VIP scores exceeding 1.0, statistical significance threshold (*p* < 0.05), and FC thresholds set at |log2(FC)| > 1.0 (equivalent to FC > 2.0 or < 0.5) (Li et al., 2021). Candidate metabolites were provisionally annotated through chromatographic (retention time) and spectrometric (*m*/*z*) matching against established databases (HMDB, http://www.hmdb.ca/) (Wishart et al., 2022) and PubChem (https://pubchem.ncbi.nlm.nih.gov/)(Kim et al., 2023), supplemented by literature verification. In addition, to achieve high-throughput and accurate compound identification, retention times, accurate molecular weights, and MS/MS fragment ions were compared against a self-constructed database containing 360 compounds (names, structures, molecular weights, formulas, and fragment ions) sourced from PubMed, ScienceDirect, and Web of Science. Additional analysis using ClpP tools and manual validation confirmed compound identities. An internal standard database improved isomer distinction and fragmentation pattern characterization, enhancing annotation accuracy. Final metabolic pattern visualization was achieved through hierarchical clustering analysis using MetaboAnalyst 5.0 (https://www.metaboanalyst.ca/) (Pang et al., 2022), presented as a colorimetric heatmap.

### 2.6 Quantification of rutin, quercetin and kaempferol by HPLC

100 mg of TLT and TOL powders were subjected to two rounds of extraction with 8.0 mL of a formic acid/methanol solution (5:95, *v*/v) at 60 °C in a water bath, with each extraction lasting 30 min. Following centrifugation at 3500 rpm for 10 min, the combined supernatant was diluted to 10.0 mL with the same formic acid/methanol solution (5:95, v/v). The supernatant was then filtered through a 0.22 μm membrane filter in preparation for UPLC analysis. Chromatographic separation was achieved using an ACQUITY UPLC HSS T3 column (150 mm × 2.1 mm, 1.8 μm, Thermo Scientific), utilizing a mobile phase composed of an acetonitrile/water (5:95, v/v) solution (eluent A) and water-0.1 % formic acid/acetonitrile (5:95, v/v) solution (eluent B). The gradient program was implemented according to a previously established method (Li, Luo, et al., 2022). For analysis, a 2 μL sample volume was injected into the column at a temperature of 30 °C. UPLC calibration curves demonstrated good linearity (R^2^ > 0.998) across tested concentrations for all standards (Table S1).

### 2.7 Quantification of main active components

Following a previously established method (Opra-Janiijevi et al., 2018), the total flavonoid content in the sample was determined. The total polyphenol content was measured using the Folin phenol method (Pérez et al., 2023). To quantify the soluble sugar and soluble protein content in TOL or TLT, the Anthrone colorimetric method and the Bradford method were employed, respectively (Zhang et al., 2024). Additionally, free amino acids were analyzed using the ninhydrin method.

### 2.8 Animal experimental design and treatment

In this study, six-week-old male ICR mice were utilized. Eight mice were maintained on a standard diet (12 % fat), while 24 mice were fed a high-fat diet (HFD, 60 % fat) for 4 weeks (diet details are shown in Table S2). Following this period, the high-fat diet group received daily injections of streptozotocin (40 mg/kg body weight/day), and the standard diet group was administered an equivalent volume of sterile sodium citrate buffer for 5 consecutive days (Winzell & Ahrén, 2004). By the 12th week, mice exhibiting fasting blood glucose levels above 11.1 mmol/L alongside impaired insulin tolerance (Fig. S2) were classified as having T2DM (Wang et al., 2022). These T2DM mice were then randomly divided into three groups: diabetic model control (MC), TOL-treated group (TOL), and TLT-treated group (TLT). The standard diet-fed mice served as the normal control (NC) group. The intervention groups (TOL and TLT) were provided with aqueous solutions of TOL and TLT, respectively, while the model group (MC) and normal control group (NC) received purified water. The tea infusions were prepared by steeping 3 g of TLT or TOL in 150 mL of boiling water for 10 min, after which the tea leaves were filtered out, and the resulting solutions were dispensed into the mice’ drinking bottles. All animal groups received daily interventions for five consecutive weeks. Prior to euthanasia, all mice were fasted overnight and humanely sacrificed via cervical dislocation under chlorate hydrate anesthesia (400 mg/kg i.p.). All experimental protocols strictly complied with the Regulations for the Administration of Affairs Concerning Experimental Animals (People's Republic of China) and followed the international 3R principles (Replacement, Reduction, Refinement) of animal welfare. This study was approved by the Animal Ethics Committee of Fuyang Normal University on November 25, 2024 (ethical approval code: FYNU2024AC010).

### 2.9 Measurement of α-glucosidase and α-amylase inhibitory activity

The inhibition assays for α-glucosidase and α-amylase were performed according to previously established methods (Guo et al., 2018), with minor adjustments. To assess α-glucosidase inhibition, a mixture containing 20 μL of TOL or TLT water extract (or 1 mM acarbose as a positive control) and 20 μL of enzyme solution (1 U/mL PBS, pH 6.8) was incubated at 37 °C. To prepare the aqueous extract, 50 g of TOL or TLT powder was boiled in 1000 mL of ultrapure water and stirred continuously at 85 °C for 30 min. Subsequently, ultrasonic extraction was performed at 75 °C for another 30 min, utilizing an average power output of 100 W. The resulting solution was then concentrated and freeze-dried to obtain the aqueous extract. Following the addition of pNPG and further incubation at 37 °C, absorbance was measured at 405 nm. The IC50 value was determined using nonlinear regression, based on the logarithmic relationship between inhibition percentage and TOL or TLT concentration. For α-amylase inhibition, a mixture of 80 μL of TOL or TLT water extract (or 1 mM acarbose) and 80 μL of enzyme solution (1 U/mL PBS, pH 6.8) was incubated at 37 °C. Subsequently, 40 μL of 1 % soluble starch PBS solution was added, and the mixture was incubated at 37 °C for 10 min. After adding DNS reagent and heating at 100 °C for 10 min, the absorbance was measured at 540 nm. α-Amylase activity was calculated as described previously (Hui et al., 2020).

### 2.10 Serum glucose and oral glucose tolerance test

Blood glucose levels were measured using the previously described method (Wu et al., 2023). A glucose tolerance test (GTT) was performed during the 5-week treatment period. Following standardized fasting protocols, mice underwent an GTT with a 2.0 g/kg body weight glucose bolus (Sigma-Aldrich, MO, USA) administered via oral gavage. Blood sampling was performed through tail vein punctures at predefined intervals (0, 30, 60, 90, 120 min post-administration). Concurrently, glycated serum protein (GSP) levels were assayed using commercial kits (Jiancheng Bio, Nanjing, China).

### 2.11 Statistical analysis

A statistical analysis was conducted using Prism 8 (GraphPad Software, USA). Differences between multiple groups or between two groups were evaluated using One-way ANOVA or *t-*tests, with a significance level of 0.05. Data are expressed as means ± SEM.

## 3 Results

### 3.1 Significant differences of metabolite profiles between TOL and TLT

**Fig. 2:**
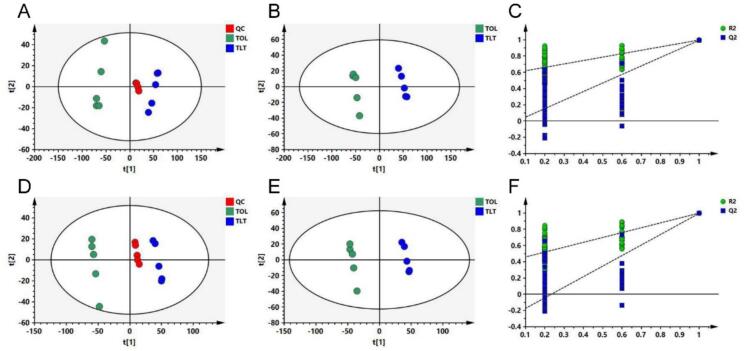
Multivariate statistical analysis between TOL (Toon leaf) and TLT (Toon leaf tea). A and D: PCA score plot of positive (R^2^X = 0.873; Q^2^ = 0.763) and negative (R^2^X = 0.958; Q^2^ = 0.777) ionization mode, respectively. B and E: PLS-DA score plot of positive (R^2^X = 0.872; R^2^Y = 0.998; Q^2^ = 0.995) and negative (R^2^X = 0.879; R^2^Y = 0.999; Q^2^ = 0.997) ionization mode, respectively. C and F: PLS-DA model validation of positive (R^2^ = 0.577; Q^2^ = -0.05) and negative (R^2^ = 0.4; Q^2^ = -0.303) ionization mode, respectively.

The chemical profiles of TOL and TLT were analyzed using UPLC Orbitrap-MS/MS. From the typical total ion current chromatograms of the samples (Fig. S3), a total of 3487 ion features were detected in positive ion mode and 2704 ion features in negative ion mode. Initially, an unsupervised PCA was performed to assess the differences between TOL and TLT. In the positive ion mode, PCA revealed that TOL samples clustered in the second and third quadrants, while TLT samples were grouped in the first and fourth quadrants (Fig. 2A). A similar pattern was observed in the negative ion mode data (Fig. 2D). Additionally, the QC samples clustered closely, demonstrating that the metabolomics analysis was stable and reliable. These findings clearly indicate that TOL and TLT have distinct chemical profiles.

Next, supervised PLS-DA statistical analysis was employed to further evaluate the differences in chemical profiles between TOL and TLT. Based on the results, TOL samples clustered in quadrants II and III, while TLT samples were grouped in quadrants I and IV (Fig. 2B, E). This further confirmed that TOL and TLT exhibit distinct chemical profiles. The model parameters, including R^2^X, R^2^Y, and Q^2^, were calculated as 0.872, 0.998, and 0.995 in the positive ion mode, and 0.879, 0.999, and 0.997 in the negative ion mode, respectively, indicating successful model establishment without overfitting. A permutation test also confirmed the absence of overfitting in both the positive and negative ion modes (Fig. 2C, F).

### 3.2 Characteristic metabolites between TOL and TLT

**Fig. 3:**
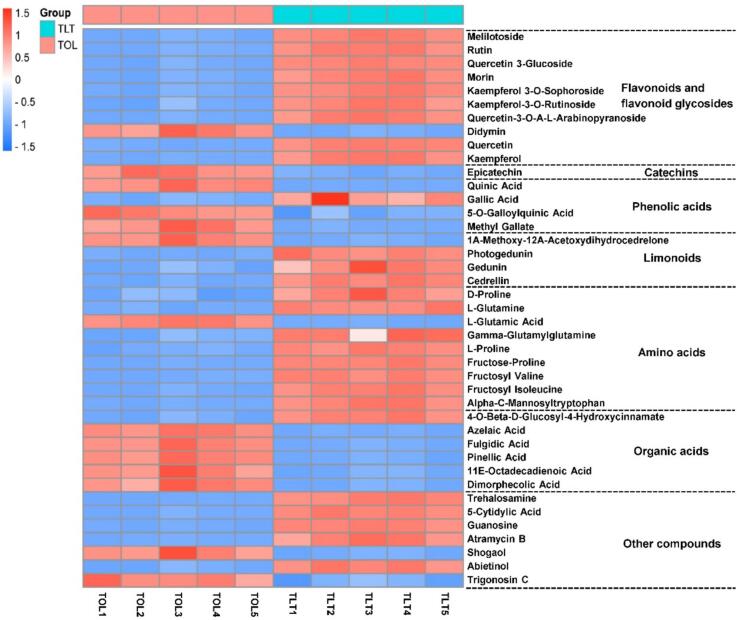
Heat-map of the metabolite contents between TOL and TLT.

A screening criterion was established to identify potential differential metabolites, requiring a VIP value greater than 1 (VIP > 1), a *p*-value in the nonparametric test less than 0.05 (*P* < 0.05), and a fold change either greater than or less than 2.0. By comparing retention time (RT), *m*/*z* values, secondary mass spectrometry fragments, various metabolome databases, and published literature, a total of forty-one differential metabolites between TOL and TLT were identified. The compounds included 10 flavonoids and flavonoid glycosides, 1 catechin, 4 phenolic acids, 4 limonoids, 9 amino acids, 6 organic acids, and 7 other compounds (Table 1). Based on the abundance of these 41 differential metabolites, a heat-map was conducted to visually summarize the differences in metabolite abundance between TOL and TLT (Fig. 3).

**Table 1:** The differentiated metabolites between TOL and TLT.

Compound Name	RT (min)	Adduct m/z	Formula	Ion mode	Fragments	VIP	*p*-Value	FC(TLT/TOL)
**Flavonoids and** **flavonoid glycosides**								
Melilotoside	9.44	325.0926	C_15_H_18_O_8_	[M-H]-	163, 119, 93	2.78	2.00 × 10^−11^	3.18
Rutin	11.51	609.146	C_27_H_30_O_16_	[M-H]-	609, 301, 300, 271, 151	8.53	1.00 × 10^−10^	3.92
Quercetin 3-Glucoside	11.85	463.0876	C_21_H_20_O_12_	[M-H]-	463, 301, 300, 255, 151	10.85	5.00 × 10^−12^	2.97
Morin	11.86	303.0497	C_15_H_10_O_7_	[M + H]+	303, 257, 229, 153, 137	5.25	1.00 × 10^−9^	3.39
Kaempferol 3-O-Sophoroside	11.93	609.146	C_27_H_30_O_16_	[M-H]-	609, 301, 300, 271, 151	1.84	5.00 × 10^−11^	4.41
Kaempferol-3-O-Rutinoside	12.13	593.1503	C_27_H_30_O_15_	[M-H]-	593, 285, 255, 227	2.84	9.00 × 10^−9^	2.14
Quercetin-3-O-Α-L-Arabinopyranoside	12.30	433.0773	C_20_H_18_O_11_	[M-H]-	433, 300, 271, 255	5.09	1.00 × 10^−9^	3.85
Didymin	14.32	593.1297	C_28_H_34_O_14_	[M-H]-	447, 285	1.01	4.00 × 10^−8^	0.34
Quercetin	14.65	301.0352	C_15_H_10_O_7_	[M-H]-	301, 178, 151, 107	4.85	5.00 × 10^−12^	11.73
Kaempferol	15.74	285.0404	C_15_H_10_O_6_	[M-H]-	285, 151, 137, 93	2.30	4.00 × 10^−10^	4.67
**Catechins**								
Epicatechin	9.52	289.0717	C_15_H_14_O_6_	[M-H]-	245, 151, 137	1.32	8.00 × 10^−9^	0.16
**Phenolic acids**								
Quinic Acid	1.38	193.0708	C_7_H_12_O_6_	[M + H]+	178, 133, 122	1.52	9.00 × 10^−6^	0.39
Gallic Acid	4.14	169.0144	C_7_H_6_O_5_	[M-H]-	125, 127, 97	1.64	1.00 × 10^−7^	2.12
5-O-Galloylquinic Acid	4.43	345.0819	C_14_H_16_O_10_	[M + H]+	153, 125	1.11	1.00 × 10^−7^	0.19
Methyl Gallate	9.38	183.03	C_8_H_8_O_5_	[M-H]-	183, 124, 78	2.95	4.00 × 10^−9^	0.43
**Limonoids**								
1Α-Methoxy-12Α-Acetoxydihydrocedrelone	17.20	307.1914	C_18_H_28_O_4_	[M-H]-	189, 235, 185, 121	3.52	1.00 × 10^−8^	0.28
Photogedunin	17.53	513.2123	C_28_H_34_O_9_	[M-H]-	513, 495	2.42	5.00 × 10^−10^	4.40
Gedunin	20.57	483.2376	C_28_H_34_O_7_	[M + H]+	423, 379, 161, 137	2.09	1.00 × 10^−5^	3.36
Cedrellin	22.40	205.1946	C_15_H_24_	[M + H]+	149, 121, 93	1.45	1.00 × 10^−7^	2.07
**Amino acids**								
D-Proline	1.60	116.0706	C_5_H_9_NO_2_	[M + H]+	70	1.03	3.00 × 10^−6^	2.28
l-Glutamine	1.23	145.0619	C_5_H_10_N_2_O_3_	[M-H]-	127, 109, 84, 41	1.77	3.00 × 10^−11^	2.76
L-Glutamic Acid	1.28	148.0604	C_5_H_9_NO_4_	[M + H]+	129, 84	3.64	1.00 × 10^−10^	0.32
Gamma-Glutamylglutamine	1.36	276.1191	C_10_H_17_N_3_O_6_	[M + H]+	147, 130	4.10	8.00 × 10^−8^	2.36
L-Proline	1.37	116.0706	C_5_H_9_NO_2_	[M + H]+	116, 70, 43	2.93	1.00 × 10^−8^	3.17
Fructose-Proline	1.41	278.1234	C_11_H_19_NO_7_	[M + H]+	260, 242, 214, 128	6.52	1.00 × 10^−9^	7.89
Fructosyl Valine	1.47	280.1391	C_11_H_21_NO_7_	[M + H]+	244, 216, 198, 118	3.34	5.00 × 10^−9^	2.95
Fructosyl Isoleucine	2.45	292.1401	C_12_H_23_NO_7_	[M-H]-	130, 101	3.12	1.00 × 10^−10^	3.58
Alpha-C-Mannosyltryptophan	7.83	365.1359	C_17_H_22_N_2_O_7_	[M-H]-	203, 116, 101	1.70	4.00 × 10^−10^	5.06
**Organic acids**								
4-O-Beta-D-Glucosyl-4-Hydroxycinnamate	9.44	344.134	C_15_H_17_O_8_	[M + NH4]+	165, 147, 119	2.74	1.00 × 10^−7^	2.92
Azelaic Acid	13.18	187.0976	C_9_H_16_O_4_	[M-H]-	187, 125, 97	1.89	4.00 × 10^−10^	0.37
Fulgidic Acid	15.31	327.2175	C_18_H_32_O_5_	[M-H]-	327, 229, 171	7.57	3.00 × 10^−9^	0.34
Pinellic Acid	15.88	329.2334	C_18_H_34_O_5_	[M-H]-	329, 229, 211, 171	4.79	4.00 × 10^−9^	0.37
11E-Octadecadienoic Acid	20.20	293.2121	C_18_H_30_O_3_	[M-H]-	275, 223, 195	6.69	1.00 × 10^−7^	0.44
Dimorphecolic Acid	20.92	295.2277	C_18_H_32_O_3_	[M-H]-	277, 195, 171	5.40	1.00 × 10^−7^	0.45
**Other compounds**								
Trehalosamine	1.21	342.1395	C_12_H_23_NO_10_	[M + H]+	324, 306, 288, 174	4.99	4.00 × 10^−11^	4.75
5-Cytidylic Acid	1.41	324.1294	C_9_H_14_N_3_O_8_P	[M + H]+	324, 112	1.53	3.00 × 10^−11^	3.54
Guanosine	3.70	282.0843	C_10_H_13_N_5_O_5_	[M-H]-	150, 133, 108	4.63	8.00 × 10^−12^	5.60
Atramycin B	8.44	451.1391	C_25_H_24_O_8_	[M-H]-	361, 289, 171, 128	1.76	2.00 × 10^−8^	6.87
Shogaol	20.05	277.2159	C_17_H_24_O_3_	[M + H]+	135, 93, 91, 79	7.99	1.00 × 10^−7^	0.36
Abietinol	22.08	289.2527	C_20_H_32_O	[M + H]+	271, 201, 149, 121, 107, 93	2.83	4.00 × 10^−7^	3.23
Trigonosin C	24.14	609.2702	C_34_H_40_O_10_	[M + H]+	609, 591	4.05	2.00 × 10^−7^	0.49

#### 3.2.1 Flavonoids and flavonoid glycosides

The flavonols and flavone glycosides are a group of compounds that are well known for their bioactive properties. The contents of ten flavonol and flavone glycosides were found to differ between TOL and TLT, as detailed in Table 1. These metabolites were identified as melilotoside, rutin, quercetin 3-glucoside, morin, kaempferol 3-O-sophoroside, kaempferol-3-O-rutinoside, quercetin-3-O-A-L-arabinopyranoside, didymin, quercetin, and kaempferol. Among these, didymin was the only compound that exhibited higher levels in TOL compared to TLT, while all other components were present in lower amounts in TOL than in TLT.

#### 3.2.2 Catechins and phenolic acids

Catechins are the primary polyphenols found in tea. In this study, we identified a catechin (epicatechin) with higher abundance in TOL than in TLT epicatechin, as illustrated in the heat map (Fig. 3). Phenolic acids, including quinic acid, gallic acid, 5-O-galloylquinic acid, and methyl gallate, were also screened and showed significant differences between TOL and TLT (Fig. 3). Specifically, quinic acid, 5-O-galloylquinic acid, and methyl gallate exhibited higher contents in TOL than in TLT. In contrast, gallic acid was present in lower amounts in TOL compared to TLT.

#### 3.2.3 Limonoids

The metabolic data revealed that four limonoids differed between TOL and TLT (Fig. 3). TLT exhibited significantly higher levels of limonoids, including photogedunin, gedunin, and edrellin, compared to TOL. However, TLT showed lower levels of 1 A-methoxy-12 A-acetoxydihydrocedrelone than TOL.

#### 3.2.4 Amino acids

Amino acids are crucial for the umami taste and aroma development of toons. Our findings demonstrated that toon leaves processed through two distinct methods exhibited differences in the levels of nine amino acids (Fig. 3), including D-proline, l-glutamine, L-glutamic acid, gamma-glutamylglutamine, L-proline, fructose-proline, fructosyl valine, fructosyl isoleucine, and alpha-C-mannosyltryptophan. With the exception of L-glutamic acid, which was more abundant in TOL than in TLT, all other amino acids were present in lower concentrations in TOL compared to TLT.

#### 3.2.5 Organic acids

Organic acids are acidic organic compounds comprised of carboxyl groups, and are widely present in plants (Chen et al., 2023). Six differential organic acids were identified between TOL and TLT: 4-O-beta-D-glucosyl-4-hydroxycinnamate, azelaic acid, fulgidic acid, pinellic acid, 11E-octadecadienoic acid, and dimorphecolic acid (Fig. 3). TLT exhibited relatively lower levels of azelaic acid, fulgidic acid, pinellic acid, 11E-octadecadienoic acid, and dimorphecolic acid compared to TOL. In contrast, the level of 4-O-beta-D-glucosyl-4-hydroxycinnamate, a single metabolite, was higher in TLT than in TOL.

#### 3.2.6 Other compounds

The metabolic analysis revealed seven differential metabolites of other compounds between TOL and TLT, including trehalosamine, 5-cytidylic acid, guanosine, atramycin B, shogaol, abietinol, and trigonosin C (Fig. 3). Trehalosamine, 5-cytidylic acid, guanosine, atramycin B, and abietinol levels were higher in TLT than in TOL. In contrast, shogaol and trigonosin C showed lower levels in TLT than in TOL.

### 3.3 Quantification of key metabolites and main active components in TOL and TLT

**Fig. 4:**
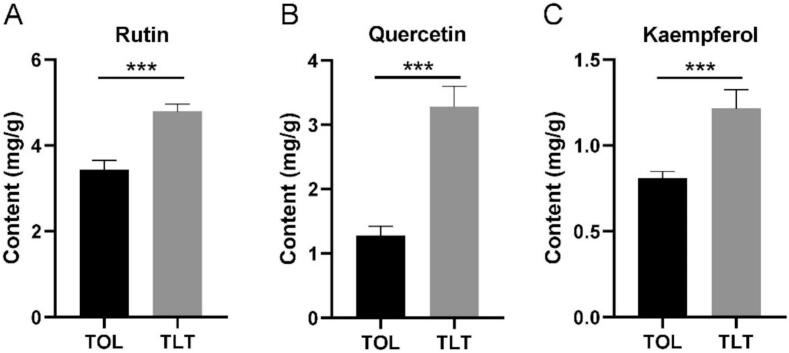
Quantification of rutin, quercetin and kaempferol between TOL and TLT. *** *p* < 0.001, data are presented as the means ± SEM (*n* = 3).

**Fig. 5:**
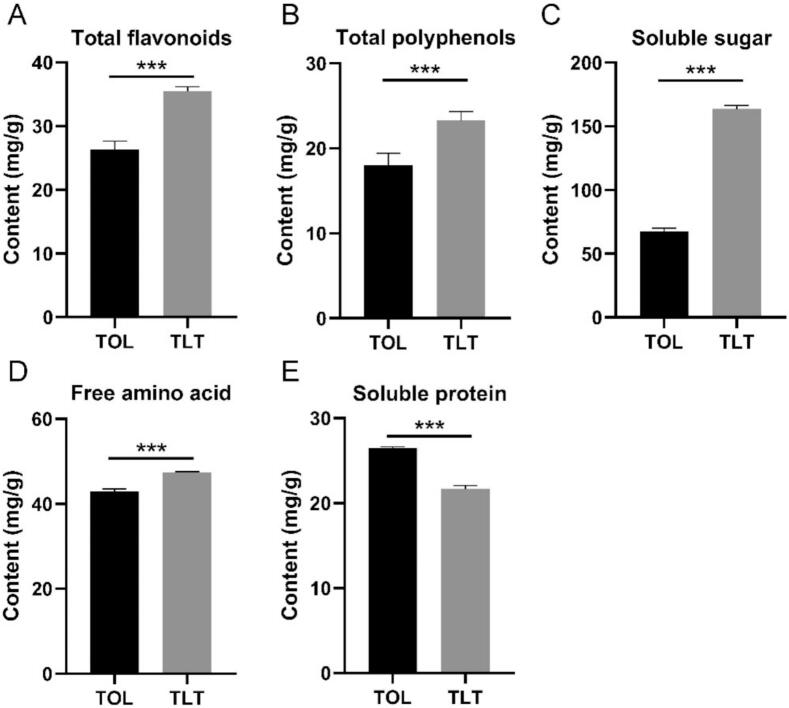
Quantitative comparison of 5 bioactive compounds in TOL and TLT. *** *p* < 0.001, data are presented as the means ± SEM (n = 3).

Untargeted metabolomics analysis revealed that TLT contained significantly higher relative amounts of flavonoids and flavonoid glycosides compared to TOL. To further validate these findings, an absolute quantitative analysis was conducted. UPLC was employed to quantify rutin, quercetin, and kaempferol using their respective standard chemicals. The quantitative results demonstrated that TLT had higher levels of rutin, quercetin, and kaempferol than TOL (Fig. 4, *p* < 0.001), which aligns with the chemical profile data. Additionally, quantitative analyses of total flavonoids, total polyphenols, soluble sugars, and free amino acids in TOL and TLT showed that TLT exhibited higher levels than TOL (Fig. 5, *p* < 0.001). However, the soluble protein content in TLT was lower than in TOL.

### 3.4 Distinct hypoglycemic effect between TOL and TLT

**Fig. 6:**
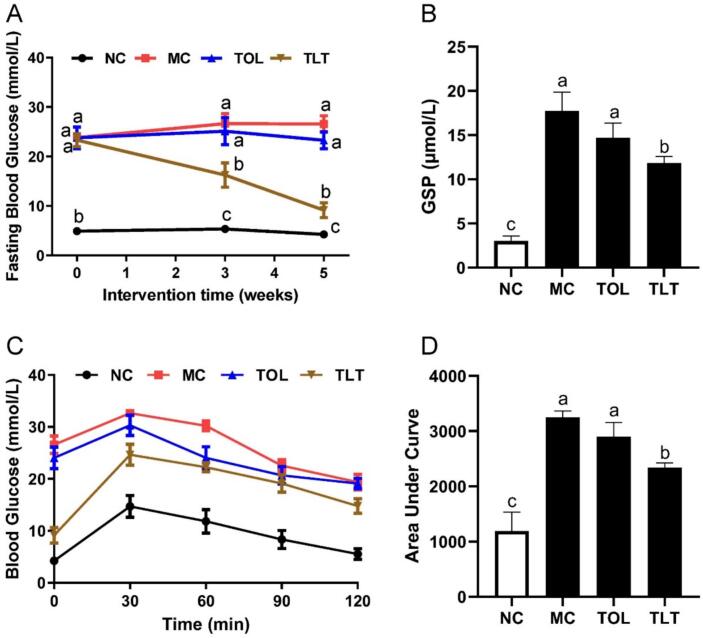
Hypoglycemic effects of TOL and TLT on diabetic model mice. (A) Blood glucose; (B) glycated serum protein (GSP) levels; (C) glucose tolerance test (GTT); (D) the glucose area under the curve. Different letters within columns denote statistically significant differences between groups (*p* < 0.05). All data are presented as means ± SEM (*n* = 8).

α-Glucosidase and α-amylase inhibitors are potential candidates for managing blood glucose levels in patients with T2DM (Proença et al., 2022). Our in vitro experiments demonstrated that the water extracts of both TOL and TLT inhibited the activity of α-glucosidase and α-amylase. However, as shown in Table 2, TLT exhibited smaller IC50 values for α-amylase and α-glucosidase compared to TOL (*p* < 0.001), indicating a stronger inhibitory effect of TLT on these enzymes. A HFD combined with streptozotocin is commonly used to induce a T2DM model. In vivo data from mice showed that after 5 weeks of high-fat feeding (week 0 of the intervention experiment), the fasting blood glucose level of the NC group was 4.27 ± 0.18 mmol/L, while the blood glucose level of mice fed a HFD exceeded 11.1 mmol/L alongside impaired insulin tolerance, confirming the successful establishment of the T2DM mouse model (Fig. S2). After 5 weeks of TOL and TLT intervention, the fasting blood glucose level of the MC group was 26.58 ± 1.68 mmol/L (exceeding 11.1 mmol/L, Fig. 6A). The TOL intervention group showed a reduction in blood glucose levels (23.28 ± 1.71 mmol/L), but the difference was not statistically significant compared to the MC group. In contrast, supplementation with the TLT significantly reduced blood glucose levels compared to the MC group (9.16 ± 1.49 mmol/L). Additionally, the GSP levels in the MC group were significantly higher than those in the NC group (*p* < 0.05), and supplementation with TLT prevented the increase in GSP levels in T2DM mice (Fig. 6B). However, supplementation with TOL did not significantly reduce GSP levels in T2DM mice.

**Table 2:** The IC50 of α-glucosidase and α-amylase in TOL and TLT.

Sample	α-glucosidase IC50 (μg/mL)	α-amylase IC50 (μg/mL)
TOL	1110.00 ± 23.66	3943.00 ± 75.10
TLT	875.30 ± 27.61***	2312.00 ± 41.20***

Note: *** *p* < 0.001, compared to the TOL.

In order to assess the regulatory effect of TLT on glucose homeostasis in T2DM mice, glucose tolerance was assessed at the conclusion of the intervention. The GTT results showed that blood glucose levels in the MC group remained above 30.0 mmol/L 60 min after glucose administration, indicating severely impaired glucose tolerance (Fig. 6C). In contrast, mice in the TOL and TLT groups exhibited lower blood glucose levels than those in the MC group, with levels decreasing after peaking at 30 min. However, the hypoglycemic effect of TOL was significantly weaker than that of TLT. Notably, the area under the curve (AUC) for blood glucose levels was remarkably decreased in the TLT group compared to the TOL group (Fig. 6D, *p* < 0.05), indicating that TLT has a stronger ability to lower blood glucose and improve glucose tolerance than TOL.

## 4 Discussion

*T. sinensis* var. ‘Heiyouchun’, a renowned cultivar of Chinese toon, has been cultivated and consumed throughout China's long history. Known for its distinctive flavor and nutritional value, toon buds and young leaves have been widely used in fermented sauces and functional foods (Jiang et al., 2019). However, mature toon leaves remain underutilized due to their pronounced bitterness and limited aromatic compounds, resulting in significant resource waste. To address this challenge, we applied black tea processing technology to produce toon leaf tea (TLT), aiming to enhance its functional properties and commercial value. Notably, systematic investigations into TLT's metabolomic fingerprint and hypoglycemic potential are still lacking in the scientific literature. Our study pioneers a comparative phytochemical analysis between TLT and conventionally dried mature toon leaves (TOL) using advanced metabolomic profiling, while also evaluating their differential hypoglycemic efficacy through integrated in vitro and in vivo pharmacological models.

Metabolomic profiling revealed 41 differentially annotated metabolites between TLT and TOL through comparative analysis. Flavonoids, which are widely distributed secondary metabolites in vascular plants, exhibit diverse biological functions (Chen et al., 2022). Mature toon leaves have reported that they are rich in flavonoids (Zhao et al., 2024). Our findings indicate that the content of flavonoids in TLT, including melilotoside, rutin, quercetin 3-glucoside, morin, kaempferol 3-O-sophoroside, kaempferol-3-O-rutinoside, quercetin-3-O-α-L-arabinopyranoside, quercetin, and kaempferol, is significantly higher than that in TOL (Fig. 3). These findings were further validated through chemometric analysis targeting three representative flavonoids: rutin, quercetin, and kaempferol (Fig. 4). This increase may be attributed to the fermentation process involved in TLT production. Traditionally, black tea fermentation was considered primarily an enzymatic oxidation process, independent of microbial activity. But in recent years, some studies have pointed out that there may be microbial activity during the processing, especially in natural fermentation, where microorganisms in the environment may participate (Liu et al., 2023; Wang et al., 2024). For instance, some research indicates that microorganisms such as *Pantoea* and *Pseudomonas* may be involved in black tea processing, potentially influencing the chemical composition and enhancing the formation of certain flavor compounds (Wang et al., 2024). Therefore, we hypothesize that the fermentation process of toon leaves also involves a combination of enzymatic oxidation and microbial fermentation. A previous study demonstrated that lactic acid bacteria fermentation increased the flavonoid content in citrus extract. Zhan et al. reported the enzymatic release of isoflavone during fermentation of natto (Zhan et al., 2024). In general, our results are consistent with those reported in those publications.

Phenolic acids, as critical astringent compounds, are important factors affecting the taste profile of tea infusions (Wen et al., 2022). Toon leaves are rich in phenolic acids. Our study identified a pronounced decrease in phenolic acid levels in TLT compared to TOL (Fig. 3). Specifically, major phenolic acids - notably quinic acid, 5-O-galloylquinic acid, and methyl gallate - showed significant reductions in TLT samples. This observation aligns with existing research trends: fermentation processes can markedly reduce the phenolic acid content (e.g., chlorogenic acid) in sunflower flour through biotransformation (Fritsch et al., 2016). Further studies suggest that microorganisms (e.g., *Aspergillus* spp.) may degrade ester-bound phenolic acids to reduce bitterness and synergistically facilitate the formation of pigments like theaflavins and thearubigins (Li, Xiao, et al., 2022). Our findings demonstrate that adopting black tea processing techniques effectively modulates phenolic acid metabolic pathways in the toon leaves, aligning with previous studies. Furthermore, organic acids—key contributors to the sourness and mellow thickness of tea infusions—exhibited significantly lower total levels in TLT compared to TOL. This suggests that fermentation may optimize the organic acid profile through microbial metabolism or enzymatic reactions, thereby enhancing the sensory quality of the final toon product.

Amino acids and their derivatives, as key taste-active metabolites in tea infusions, play a central regulatory role in the sensory characteristics of tea extracts and serve as core regulatory factors in shaping the complexity of tea flavor. This study revealed that while black tea processing techniques significantly reduce the soluble protein content in toon leaves, they effectively increase the total amino acid content (Fig. 5). This compositional change contributes to enhancing the flavor expression of toon leaf tea. Consistent with previous research reports, the withering process in the processing of black tea promotes the hydrolysis of proteins into free amino acids (Zhao et al., 2023), and this study similarly observed a significant increase in the free amino acid content of toon leaves after withering. This discovery not only deepens our understanding of the transformation mechanisms of flavor compounds during tea processing but also provides a scientific basis for the regulation of tea flavor.

Limonoids, a distinctive class of tetracyclic triterpenoid derivatives characterized by their unique furanolactone structure, have been identified as key bioactive constituents in *T. sinensis* (Fu et al., 2020). Emerging evidence suggests these phytochemicals demonstrate a broad spectrum of pharmacological activities. Notably, Fu et al. have documented the occurrence of limonoids in fresh young leaves and shoots of toon, revealing their potential neuroprotective efficacy (Fu et al., 2020). Subsequent investigations by Hu et al. systematically investigated the bioactivity profile of toon-derived limonoids, demonstrating significant free radical scavenging capacity, anti-inflammatory effects through NF-κB pathway inhibition, and selective cytotoxicity against HepG2 hepatocarcinoma cells (Hu et al., 2016). Our research shows that black tea fermentation processing induces an enhancement in limonin biotransformation compared to conventional direct-drying preservation methods, suggesting enzymatic activation during the oxidative fermentation stage substantially enhances limonoid metabolism in mature toon leaves.

Diabetes is a chronic metabolic disorder syndrome characterized by persistent hyperglycemia. Recently, natural products have attracted significant scientific attention due to their potential for diabetes management. Research has demonstrated the remarkable value of toon leaf (TL) components in glycemic regulation: Non-polar fractions extracted via supercritical carbon dioxide can alleviate the progression of type 2 diabetes mellitus and hepatic fibrosis in streptozotocin-induced diabetic mice by modulating lipid metabolism (Hsieh et al., 2012), while aqueous extracts enhance glucose metabolism in alloxan-induced diabetic models through upregulation of glucose transporter 4 (GLUT4) expression (Wang et al., 2008). Notably, specific phytochemicals isolated from TL, such as rutin and quercetin, have been shown to improve glucose uptake via insulin receptor kinase (IRK) activation (Hsu et al., 2014) and suppress inflammatory pathways in HFD models (Zhang et al., 2016), respectively. While prior research has established the hypoglycemic potential of TL, the influence of processing methodologies on its therapeutic efficacy remains insufficiently investigated. Our findings present the first demonstration that black tea-fermented TLT exhibits superior hypoglycemic activity compared to traditionally dried TOL, as evidenced by enhanced inhibition of α-amylase and α-glucosidase enzymes in vitro, accompanied by marked improvements in glucose tolerance in murine models (Fig. 6). Metabolomic analysis revealed a significant elevation in gallic acid, rutin, quercetin, and limonoid compounds within TLT, which may contribute to its augmented antidiabetic properties. This processing-induced compositional change introduces a novel strategy for developing multitarget botanical antidiabetic agents, potentially addressing the limitations of current single-target therapeutics.

## 5 Conclusion

In summary, this study presents the first comprehensive metabolite profile of TLT and highlights its differences compared to TOL. Our findings reveal that TLT contains significantly higher levels of flavonoids, flavonoid glycosides, limonoids, and amino acids than TOL, which likely contributes to its enhanced bioactivity and distinct flavor profile. Furthermore, both in vitro α-glucosidase/α-amylase inhibition assays and in vivo animal studies demonstrate that TLT possesses superior hypoglycemic effects compared to TOL. This research not only clarifies the chemical composition differences in mature toon leaves resulting from two different processing methods but also offers a scientific foundation for the resource utilization of mature toon leaves.

## CRediT authorship contribution statement

**Guohuo Wu:** Writing – original draft, Methodology, Investigation, Funding acquisition, Data curation, Conceptualization. **Shuolei Xu:** Writing – original draft, Methodology, Investigation. **Zhaoyun Chen:** Writing – original draft, Methodology, Investigation. **Li Wu:** Methodology, Investigation. **Wenli Fan:** Software, Data curation. **Xiaoyun Wu:** Investigation, Data curation. **Mengyu Li:** Software, Data curation. **Changqing Qu:** Supervision, Formal analysis. **Yuntao Ji:** Writing – review & editing, Supervision, Conceptualization.

## Declaration of competing interest

The authors declare that they have no known competing financial interests or personal relationships.

that could have appeared to influence the work reported in this paper.

## Data Availability

Data will be made available on request.

## Appendix A Supplementary data

Supplementary material Image 1
