# Supplementary material for: Comparative analysis of metabolite signatures and hypoglycemic effects in Toona sinensis leaves processed by two distinct methods
Source: Food Chem X. 2025 Jul 20;29:102821. doi: 10.1016/j.fochx.2025.102821 (PMC12309930; doi:10.1016/j.fochx.2025.102821)
Supplement: Supplementary file 1 — Supplementary material [file mmc1.docx]

**Table S1** Linear equations and correlation coefficients (R^2^) of rutin, quercetin and kaempferol.

| Compound | Linear equation | R^2^ |
| --- | --- | --- |
| Rutin | y = 43305.6x - 7646 | 0.998 |
| Quercetin | y = 27489.2x - 7417.2 | 0.999 |
| Kaempferol | y = 23661.0x - 3687.6 | 0.998 |

Note: x, amount of each analyzed injection compound (µg/mL); y, peak area.

**Table S2.** The composition of the experimental diets

| **Ingredient (g)** | **low-fat diet** | **high-fat diet** |
| --- | --- | --- |
| Casein | 191 | 262 |
| Cornstarch | 484 | 0 |
| Dextrin | 120 | 164 |
| Sucrose | 66 | 90 |
| Soybean oil | 24 | 33 |
| Lard | 19 | 321 |
| Cellulose | 48 | 65 |
| Mineral mix | 33 | 46 |
| Vitamin mix | 10 | 13 |
| L-Cystine | 3 | 4 |
| Line bitartrate | 2 | 3 |
| TBHQ | 0.01 | 0.07 |
| Total | 1000 | 1000 |
| Total energy |  |  |
| Protein, % | 20.6 | 19 |
| Fat, % | 12.0 | 60 |
| Carbohydrate, % | 67.4 | 21 |
| Energy, Kcal/g | 3.616 | 5.2 |

Figure S1


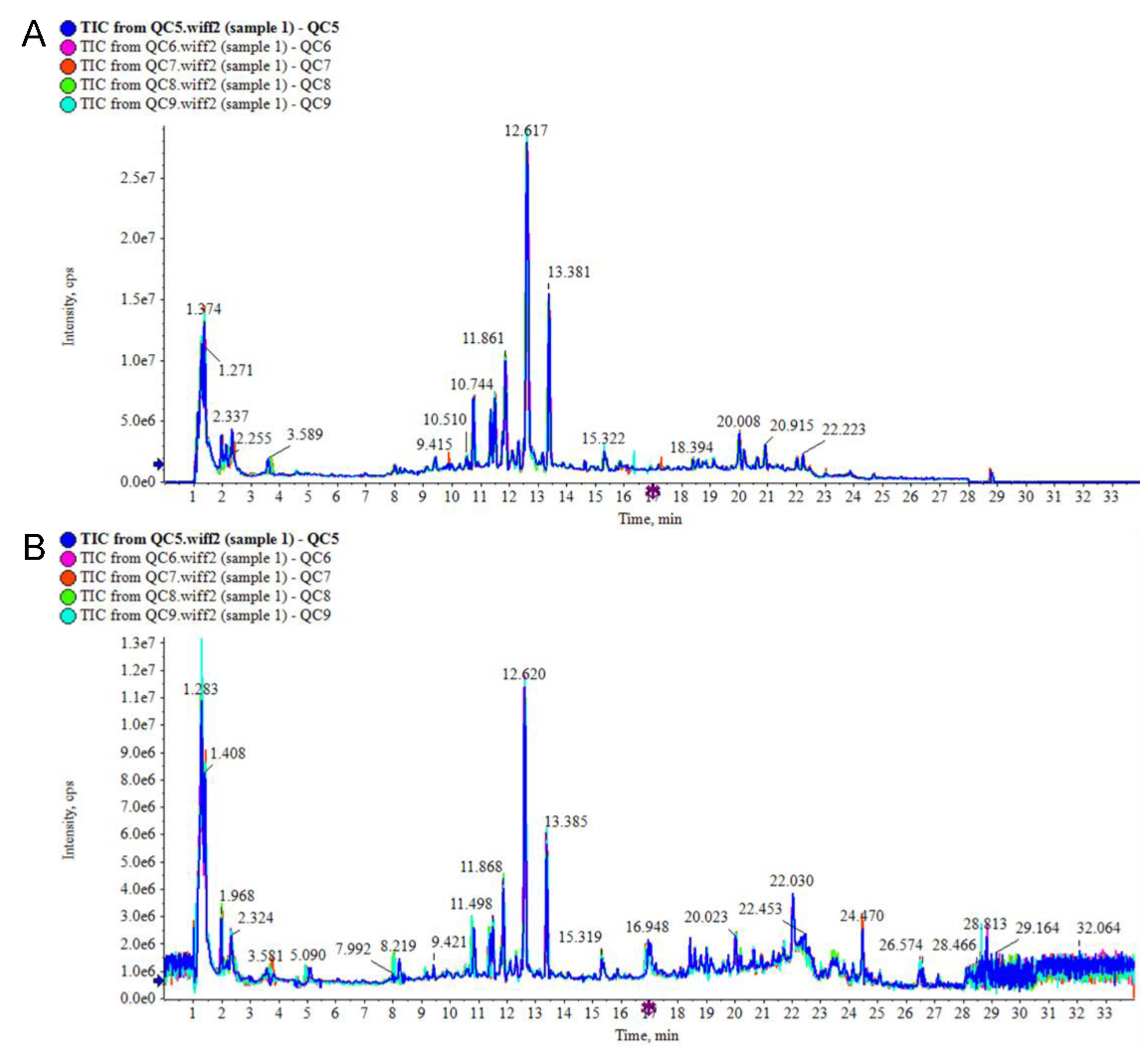


Figure S1 TIC overlay chromatograms of QC samples in positive and negative ion modes. (A) Negative ion mode; (B) positive ion mode.

Figure S2


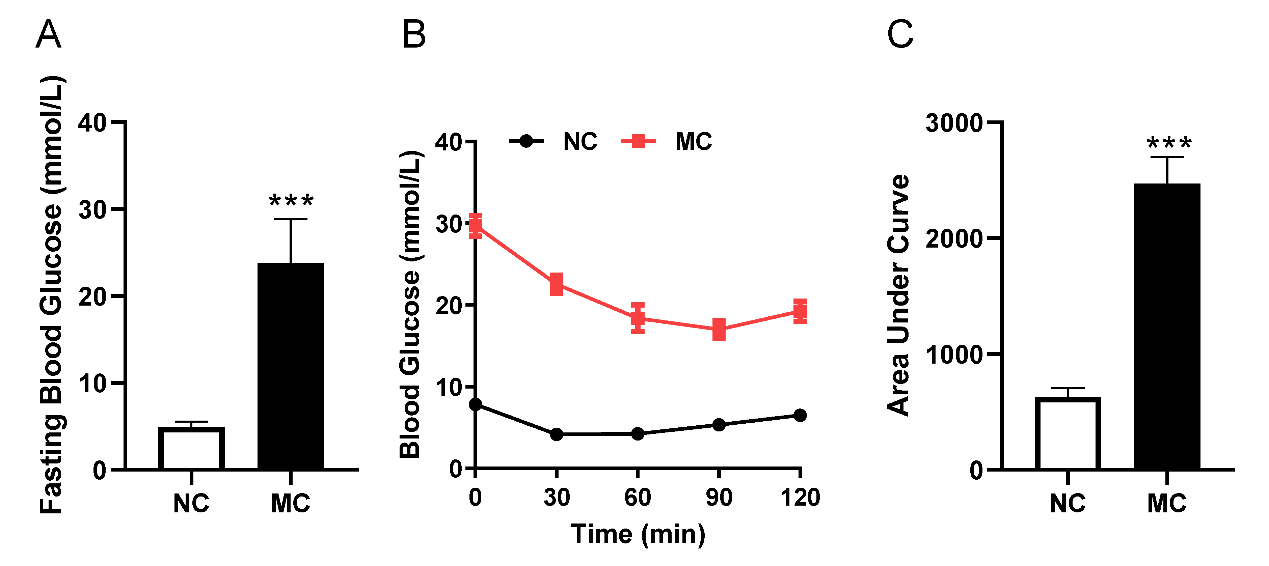


Figure S2 Analysis of fasting blood glucose and insulin tolerance in T2DM mouse model. (A) Fasting blood glucose; (B) insulin tolerance test (ITT); (C) the glucose area under the curve. *** *p* < 0.001.

Figure S3


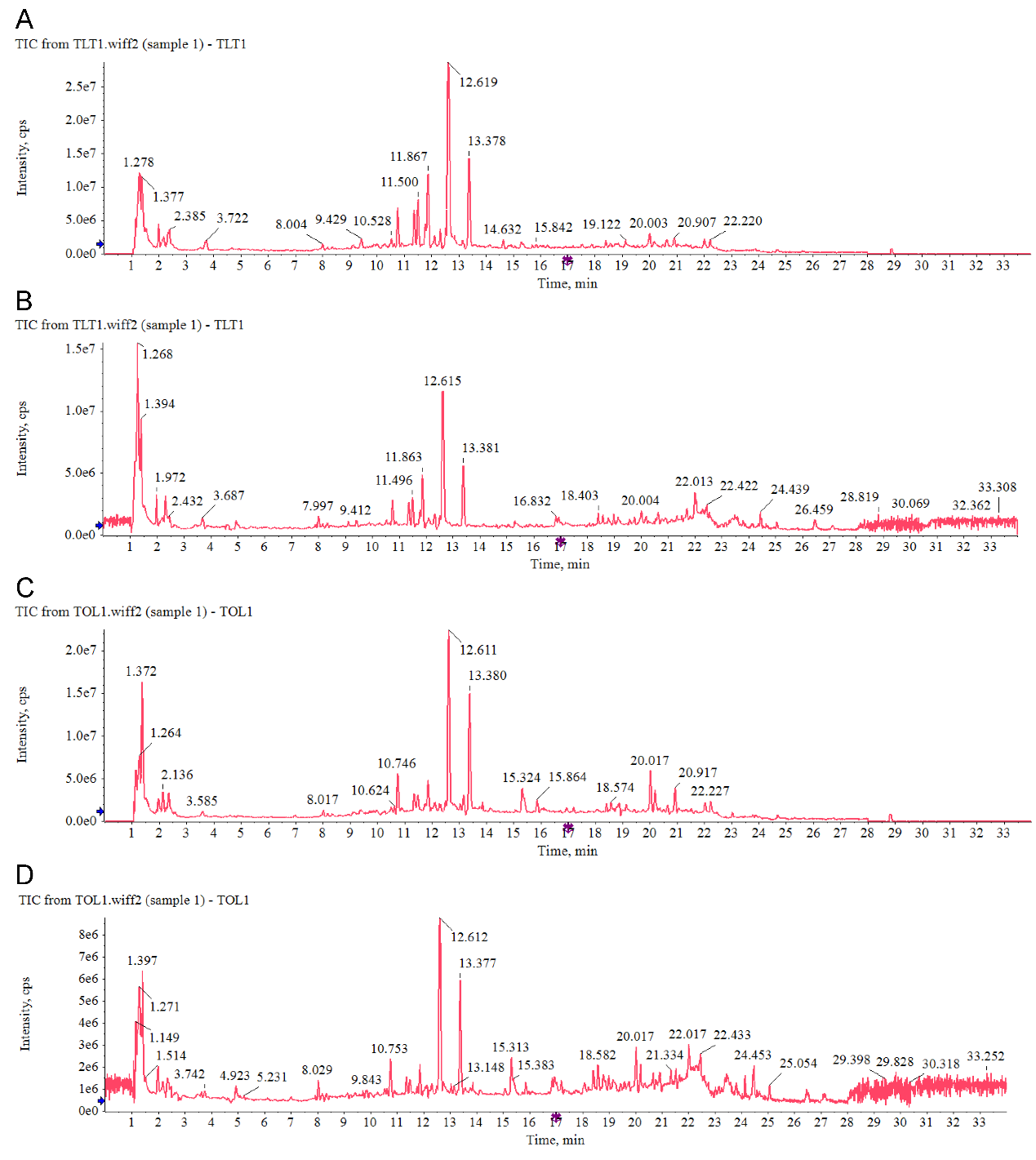


Figure S3 TIC overlay chromatograms of samples in positive and negative ion modes. (A, B) TIC overlay chromatograms of TLT in negative ion mode and positive ion mode; (C, D) TIC overlay chromatograms of TLT in negative ion mode and positive ion mode.
